# Supplementary material for: Growth Score: a single metric to define growth in 96-well phenotype assays
Source: PeerJ. 2018 Apr 19;6:e4681. doi: 10.7717/peerj.4681 (PMC5911387; doi:10.7717/peerj.4681)
Supplement: Supplemental Information 3 — The PDF version of the growth curve simulation Jupyter Notebook. [file peerj-06-4681-s003.pdf]

Growth Score: a single metric to define growth in 96-well phenotype assays

Author: Daniel A. Cuevas

Date: January 22, 2018

Import Python libraries

In [1]:
```
%matplotlib inline
```

In [2]:
```
import matplotlib.pyplot as plt
import numpy as np
import pandas as pd
import seaborn as sns
```

In [3]:
```
print("Pandas:", pd.__version__)
print("Seaborn:", sns.__version__)
print("NumPy:", np.__version__)
```

```
Pandas: 0.20.2
Seaborn: 0.8.1
NumPy: 1.13.1
```

Simulate parameters

In [4]:
```
# Generate random lists of data
N = 1000  # Number of curves
t = 50  # N hours
np.random.seed(9)
y0 = np.random.uniform(0.05, 0.10, N)
A = np.random.uniform(0.1, 1.2, N)
mu = [np.random.uniform(a / t, a * 1.1, 1)[0] for a in A]
lag = np.random.uniform(0, 20, N)
sample = ["sample{}".format(x) for x in range(N)]
time = np.arange(start=0, stop=t, step=0.5)
```

In [5]:
```
pheno = pd.DataFrame({"sample": sample, "y0": y0, "A": A, "mu": mu, "la
g": lag})
```

Simulate growth curves from parameters

In [6]:
```
def logistic(time, y0, lag, mu, A):
    logcurve = []
    logcurve = y0 + ((A - y0) / (1 + np.exp(((4 * mu / A) * (lag - time
)) + 2)))
    return logcurve
```

In [7]:
```
curve_data = {"sample": [], "time": [], "od": []}
for idx, x in pheno.iterrows():
    sample = x["sample"]
    y0 = x["y0"]
    lag = x["lag"]
    mu = x["mu"]
    A = x["A"]
    logcurve = logistic(time, y0, lag, mu, A)
    for i, t in enumerate(time):
        curve_data["sample"].append(sample)
        curve_data["time"].append(t)
        curve_data["od"].append(logcurve[i])
curves = pd.DataFrame(curve_data)
```

Calculate GS

In [8]:
```
pheno["growthscore"] = (pheno["A"] - pheno["y0"]) + pheno["mu"] * 0.25
```

Calculate GL

In [9]:
```
def GL(logcurve, y0, A):
    return len(logcurve) / np.sum((1 / ((A - y0) + (logcurve - y0))))
```

In [10]:
```
growthlevels = {"sample" :[], "growthlevel": []}
for name, group in curves.groupby("sample"):
    logcurve = np.array(group["od"].values)
    y0 = pheno.loc[pheno["sample"] == name, "y0"].values[0]
    A = pheno.loc[pheno["sample"] == name, "A"].values[0]
    growthlevels["sample"].append(name)
    growthlevels["growthlevel"].append(GL(logcurve, y0, A))
tmp_df = pd.DataFrame(growthlevels)
tmp_df.set_index("sample", inplace=True)
```

In [11]:
```
data = pheno.join(tmp_df, on="sample")
```

GL and GS thresholds

In [12]:

```
data["GL class"] = "--"
data.loc[data["growthlevel"] > 0.25, "GL class"] = "-"
data.loc[data["growthlevel"] > 0.35, "GL class"] = "+"
data.loc[data["growthlevel"] > 0.50, "GL class"] = "++"
data.loc[data["growthlevel"] > 0.75, "GL class"] = "+++"
data.loc[data["growthlevel"] > 1.00, "GL class"] = "++++"
data["GS class"] = "--"
data.loc[data["growthscore"] > 0.15, "GS class"] = "-"
data.loc[data["growthscore"] > 0.25, "GS class"] = "+"
data.loc[data["growthscore"] > 0.35, "GS class"] = "++"
data.loc[data["growthscore"] > 0.50, "GS class"] = "+++"
data.loc[data["growthscore"] > 0.65, "GS class"] = "++++"
```

In [13]:

```
sns.set(style='white', context='talk')
g = sns.jointplot("growthlevel", "growthscore", data=data, kind="reg", s
ize=8)
g.ax_joint.collections[0].set_visible(False)
colors = sns.color_palette()
for idx, row in data.iterrows():
    if row["GL class"] == "--":
        g.ax_joint.plot(row["growthlevel"], row["growthscore"], color=co
lors[3], marker="o", markersize=5)
    elif row["GL class"] == "-":
        g.ax_joint.plot(row["growthlevel"], row["growthscore"], color=co
lors[5], marker="o", markersize=5)
    elif row["GL class"] == "+":
        g.ax_joint.plot(row["growthlevel"], row["growthscore"], color=co
lors[4], marker="o", markersize=5)
    elif row["GL class"] == "++":
        g.ax_joint.plot(row["growthlevel"], row["growthscore"], color=co
lors[2], marker="o", markersize=5)
    elif row["GL class"] == "+++":
        g.ax_joint.plot(row["growthlevel"], row["growthscore"], color=co
lors[1], marker="o", markersize=5)
    else:
        g.ax_joint.plot(row["growthlevel"], row["growthscore"], color=co
lors[0], marker="o", markersize=5)

# Draw lines
xlim = plt.xlim(-0.1, None)
ylim = plt.ylim(-0.1, None)
g.ax_joint.plot(xlim, [0.15, 0.15], "k--", linewidth=1)
g.ax_joint.plot(xlim, [0.25, 0.25], "k--", linewidth=1)
g.ax_joint.plot(xlim, [0.35, 0.35], "k--", linewidth=1)
g.ax_joint.plot(xlim, [0.50, 0.50], "k--", linewidth=1)
g.ax_joint.plot(xlim, [0.65, 0.65], "k--", linewidth=1)
txt = plt.xlabel("Growth Level")
txt = plt.ylabel("Growth Score")
```

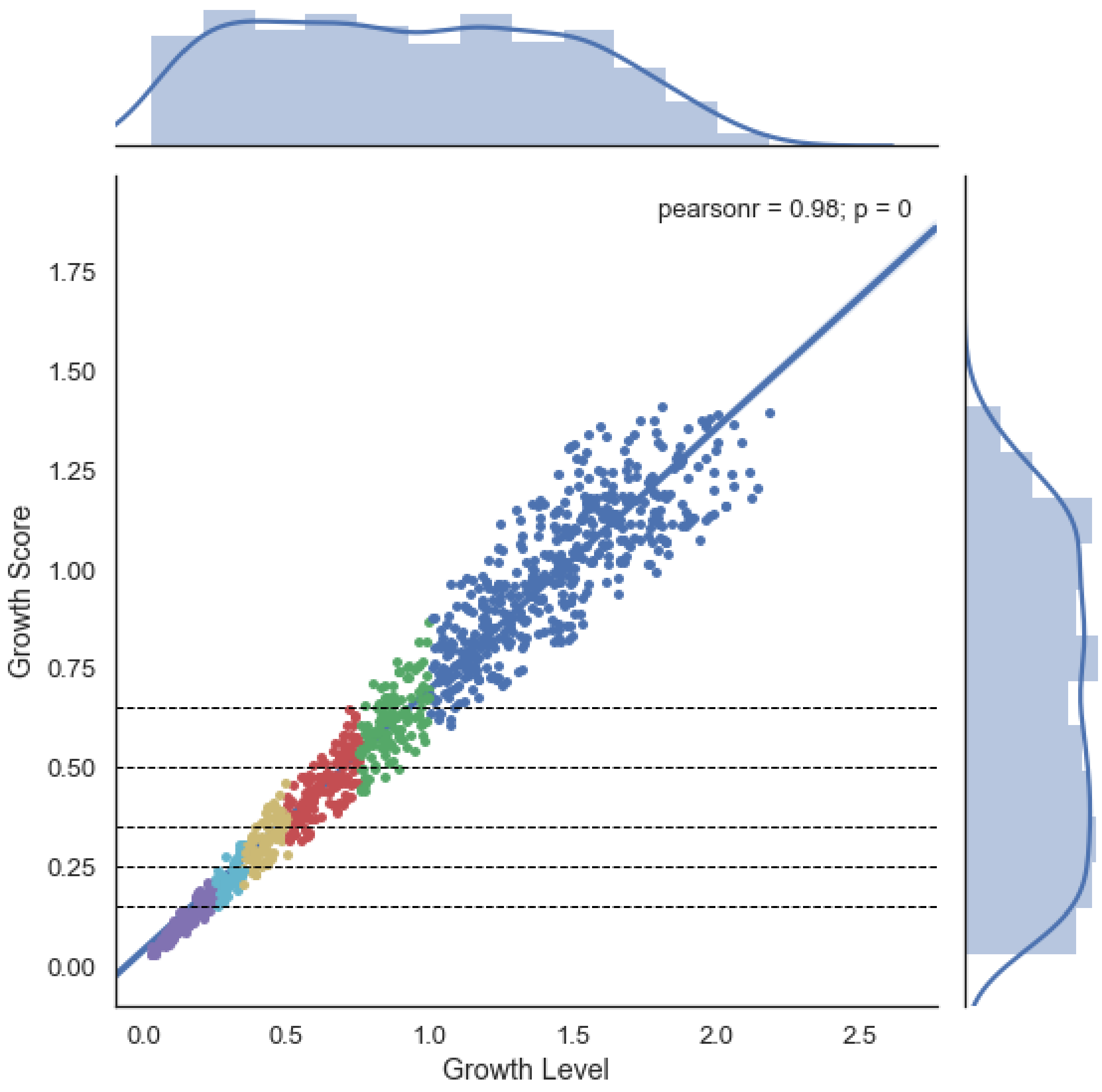

In [14]:
```
g.savefig("figure1.pdf", dpi=400, bbox_inches="tight")
```

The regression plot above illustrates the strong congruency between GL and GS. Pearson Correlation Coefficient $\rho = 0.98$.

In [15]:
```
curves_plot = curves.join(data[["sample", "GL class", "GS class"]].set_i
ndex("sample"), on="sample")
```

In [16]:
```
curves_plot["GL class"] = curves_plot["GL class"].astype("category")
curves_plot["GL class"].cat.reorder_categories(["++++", "+++", "++", "+"
, "-", "--"], inplace=True)
curves_plot["GS class"] = curves_plot["GS class"].astype("category")
curves_plot["GS class"].cat.reorder_categories(["++++", "+++", "++", "+"
, "-", "--"], inplace=True)
```

In [17]:
```
ax = sns.tsplot(data=curves_plot, time="time", unit="sample", condition=
"GL class", value="od", err_style="boot_traces", n_boot=100)
sns.despine()
```

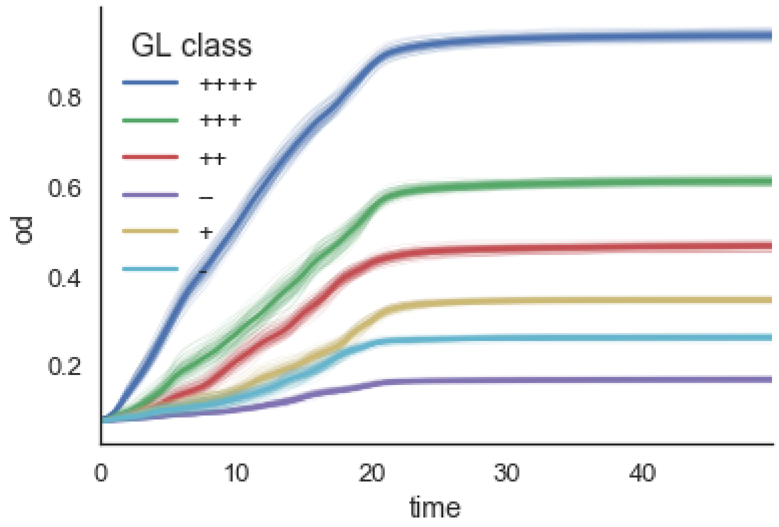

In [18]:
```
ax.figure.savefig("figure2A.pdf", dpi=400, bbox_inches="tight")
```

In [19]:
```
ax = sns.tsplot(data=curves_plot, time="time", unit="sample", condition=
"GS class", value="od", err_style="boot_traces", n_boot=100)
sns.despine()
```

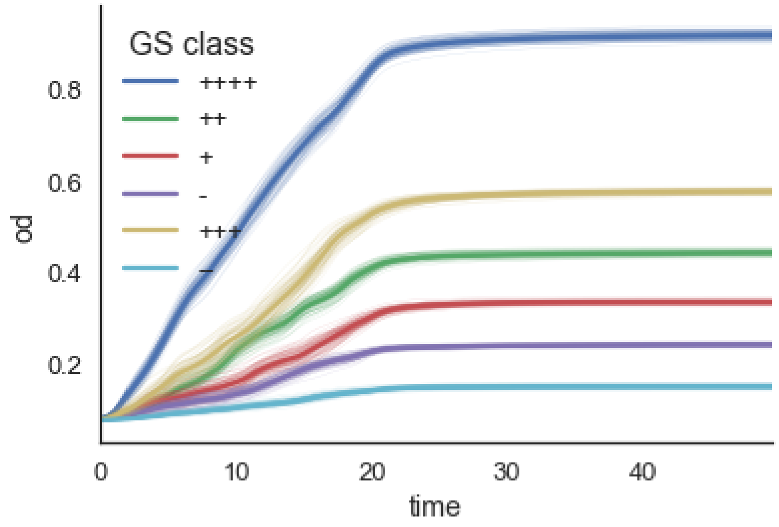

In [20]:
```
ax.figure.savefig("figure2B.pdf", dpi=400, bbox_inches="tight")
```
